# Use of thyroid hormones in euthyroid patients with unexplained fatigue: analyses of aggregate data from European national surveys of professional endocrine society members

**DOI:** 10.3389/fendo.2025.1713814

**Published:** 2025-11-05

**Authors:** Tomasz Bednarczuk, Aleksandra Sugier, Roberto Attanasio, Endre V. Nagy, Roberto Negro, Enrico Papini, Miloš Žarković, Ersin Akarsu, Maria Alevizaki, Göksun Ayvaz, Biljana Nedeljković Beleslin, Eszter Berta, Miklos Bodor, Anna M. Borissova, Mihail Boyanov, Camille Buffet, Maria-Cristina Burlacu, Chagit A. Cohen, Jasmina Ćirić, Juan J. Díez, Harald Dobnig, Valentin Fadeyev, Benjamin C. T. Field, Eric Fliers, Dagmar Führer-Sakel, Jacob S. Frølich, Juan C. Galofré, Tommi Hakala, Jan Jiskra, Peter A. Kopp, Michael Krebs, Michal Kršek, Martin Kužma, Ivica Lazúrová, Laurence Leenhardt, Stephen Ludgate, Vitaliy Luchytskiy, Anne McGowan, Miguel Melo, Saara Metso, Carla Moran, Tatyana Morgunova, Dan A. Niculescu, Božidar Perić, Tereza Planck, Catalina Poiana, Francisca M. Puga, Eyal Robenshtok, Patrick Rosselet, Marek Ruchala, Kamilla R. Riis, Alla Shepelkevich, Mykola D. Tronko, David Unuane, Irfan Vardarli, W. Edward Visser, Andromachi Vryonidou, Younes Ramazan Younes, Elena Yurenya, Petros Perros, Laszlo Hegedüs

**Affiliations:** ^1^ Department of Internal Medicine and Endocrinology, Medical University of Warsaw, Warsaw, Poland; ^2^ Scientific Committee Associazione Medici Endocrinologi, Udine, Italy; ^3^ Division of Endocrinology, Department of Medicine, Faculty of Medicine, University of Debrecen, Debrecen, Hungary; ^4^ Department of Experimental Medicine, University of Salento and Division of Endocrinology V. Fazzi Hospital, Lecce, Italy; ^5^ Department of Endocrinology and Metabolism, Regina Apostolorum Hospital, Lifenet Health Group, Albano Laziale, Rome, Italy; ^6^ Faculty of Medicine, University of Belgrade, Belgrade, Serbia; ^7^ Department of Internal Medicine, Division of Endocrinology, Faculty of Medicine, Gaziantep University, Gaziantep, Türkiye; ^8^ Endocrine Unit and Diabetes Centre, Department of Clinical Therapeutics, Alexandra Hospital, School of Medicine, National and Kapodistrian University of Athens, Athens, Greece; ^9^ Department of Endocrinology and Metabolism, Koru Ankara Hospital, Ankara, Türkiye; ^10^ Clinic of Endocrinology and Metabolism, University Hospital “Sofiamed”, Medical Faculty, Sofia University “Saint Kliment Ohridski”, Sofia, Bulgaria; ^11^ Clinic of Endocrinology and Metabolism, University Hospital “Alexandrovska”, Sofia, Bulgaria; ^12^ Department of Internal Medicine, Medical University, Sofia, Bulgaria; ^13^ Sorbonne Universitè, GRC n 16, GRC Thyroid Tumors, Thyroid Diseases and Endocrine Tumor Department, AP-HP, Hôpital Pitié-Salpêtriére, Paris, France; ^14^ Department of Endocrinology and Nutrition, Cliniques Universitaires St-Luc, Université Catholique de Louvain, Brussels, Belgium; ^15^ Rabin Medical Center, Tel-Aviv University, Tel-Aviv, Israel; ^16^ Department of Endocrinology, Hospital Universitario Puerta de Hierro Majadahonda, Madrid, Spain; ^17^ Instituto de Investigación Sanitaria Puerta de Hierro Segovia de Arana, Madrid, Spain; ^18^ Department of Medicine, Universidad Autónoma de Madrid, Madrid, Spain; ^19^ Thyroid Practice and Specialty Practice for Thyroid Radiofrequency Ablation, Kumberg, Austria; ^20^ Department of Endocrinology No. 1, N.V. Sklifosovsky Institute of Clinical Medicine, I.M. Sechenov First Moscow State Medical University, Moscow, Russia; ^21^ Section of Clinical Medicine, Faculty of Health and Medical Sciences, University of Surrey, Guildford, United Kingdom; ^22^ Department of Endocrinology and Metabolism, Amsterdam UMC, University of Amsterdam, Amsterdam, Netherlands; ^23^ Department of Endocrinology, Diabetes and Metabolism, University Hospital Essen, University of Amsterdam, Essen, Germany; ^24^ Department of Endocrinology, Odense University Hospital, Odense, Denmark; ^25^ Department of Endocrinology, Clínica Universidad de Navarra, Pamplona, Spain; ^26^ Instituto de Investigación Sanitaria de Navarra, Pamplona, Spain; ^27^ Department of Surgery, Tampere University Hospital, Tampere, Finland; ^28^ 3^rd^ Department of Medicine, 1^st^ Faculty of Medicine, Charles University, General University Hospital, Prague, Czechia; ^29^ Division of Endocrinology, Diabetology and Metabolism, University of Lausanne, Lausanne, Switzerland; ^30^ Internal Medicine III, Division of Endocrinology, Medical University of Vienna, Vienna, Austria; ^31^ 5^th^ Department of Internal Medicine, Medical Faculty of Comenius University and University Hospital, Bratislava, Slovakia; ^32^ P. J. Šafárik University Košice, 1^st^ Department of Internal Medicine of the Medical Faculty, Košice, Slovakia; ^33^ The School of Medicine, Trinity College Dublin, The University of Dublin, Dublin, Ireland; ^34^ Robert Graves Institute, Tallaght University Hospital, Dublin, Ireland; ^35^ Department of Reproductive Endocrinology, Institute of Endocrinology and Metabolism named after V.P. Komissarenko, National Academy of Medical Science of Ukraine, Kyiv, Ukraine; ^36^ Department of Endocrinology, Diabetes and Metabolism Coimbra Local Health Unit, Medical Faculty, University of Coimbra, Coimbra, Portugal; ^37^ Diabetes and Endocrinology Section, Beacon Hospital, Dublin, Ireland; ^38^ School of Medicine, University College Dublin, Dublin, Ireland; ^39^ Endocrine Department, St Vincent’s University Hospital, Dublin, Ireland; ^40^ Department of Endocrinology, Carol Davila University of Medicine and Pharmacy, Bucharest, Romania; ^41^ Department of Endocrinology, Diabetes and Metabolic Diseases “Mladen Sekso”, University Hospital Center “Sisters of Mercy”, Zagreb, Croatia; ^42^ Department of Endocrinology, Skåne University Hospital, Malmö, Sweden; ^43^ Serviço de Endocrinologia, Diabetes e Metabolismo, ULS São João, Porto, Portugal; ^44^ Endocrinology Institute, Rabin Medical Center, Gray Faculty of Medicine, Tel Aviv University, Tel-Aviv, Israel; ^45^ Cabinet Médical 2, Rue Bellefontaine, Lausanne, Switzerland; ^46^ Department of Endocrinology, Metabolism and Internal Medicine, Poznan University of Medical Sciences, Poznan, Poland; ^47^ Department of Endocrinology, Belarusian State Medical University, Minsk, Belarus; ^48^ V. P. Komissarenko Institute of Endocrinology and Metabolism, National Academy of Medical Science of Ukraine, Kyiv, Ukraine; ^49^ Department of Internal Medicine, Endocrine Unit, UZ Brussel, Vrije Universiteit Brussel, Brussels, Belgium; ^50^ Department of Medicine I, Klinikum Vest GmbH Knappschaftskrankenhaus Recklinghausen, Academic Hospital, Ruhr-University Bochum, Recklinghausen, Germany; ^51^ 5^th^ Medical Department, Division of Endocrinology and Diabetes, Medical Faculty Mannheim, Heidelberg University, Mannheim, Germany; ^52^ Rotterdam Thyroid Center, Department of Internal Medicine, Erasmus MC, Rotterdam, Netherlands; ^53^ Department of Endocrinology and Diabetes Centre, Hellenic Red Cross Hospital, Athens, Greece; ^54^ East Surrey Hospital, Surrey and Sussex Healthcare NHS Trust, Redhill, United Kingdom; ^55^ Minsk Endocrinology Medical Center, Minsk, Belarus; ^56^ Translational and Clinical Research Institute, Newcastle University, Newcastle upon Tyne, United Kingdom

**Keywords:** euthyroidism, fatigue, levothyroxine, thyroid hormone, survey

## Abstract

**Background:**

Managing patients with fatigue is a clinical challenge. Because fatigue is often reported in hypothyroidism, thyroid hormone (TH) therapy may sometimes be incorrectly considered for biochemically euthyroid individuals. This study aimed to evaluate the prevalence and determinants of this practice in different European countries.

**Methods:**

We analyzed aggregate data from the THESIS (Treatment of Hypothyroidism in Europe by Specialists: an International Survey) online survey. We analyzed responses from 5,695 members of 28 national endocrine/thyroid societies’ specialists to the statement: “Thyroid hormones may be indicated in biochemically euthyroid patients with unexplained fatigue”.

**Results:**

Overall, 7.5% (426/5695) of respondents indicated that TH therapy might be considered for euthyroid patients with unexplained fatigue. The proportion of positive responses varied widely across different countries (between 1.1% in Switzerland and 29.3% in Serbia; p=2 ×10^-16^) and regions (between 4.7% in Western Europe and 8.7% in Western Asia or 8.8% in Eastern Europe; p=0.004). TH were more frequently prescribed for unexplained fatigue by male respondents (Odds Ratio, OR 1.45, 95% CI 1.18-1.78) and physicians practicing in private practice (OR 1.27, 95% CI 1.02-1.58), and less frequently by endocrinologists (OR 0.62, 95% CI 0.46-0.83).

**Conclusion:**

A small, yet not negligible percentage of European thyroid-focused physicians consider using TH for euthyroid patients with unexplained fatigue, with significant variations based on geographic, demographic, and practice-related factors. Using levothyroxine and/or liothyronine in such cases lacks evidence and may partially contribute to the concerning overuse of TH therapy.

## 1 Introduction

Fatigue is among the most frequently reported symptoms of overt hypothyroidism, potentially causing significant impairment in daily activities and severely impacting patients’ quality of life (QoL) (1–4). Fatigue is reported across all age groups in hypothyroid patients, with a prevalence reaching 95% in individuals under 50 years old and its severity correlates with the decrease in serum thyroxine (T4) levels (5, 6). Although thyroid hormones play a pivotal role in maintaining neuronal integrity and function in the adult brain, the pathophysiology underlying hypothyroidism-related fatigue remains largely unknown (7, 8). It is speculated that: (i) fatigue results from a substantial decrease in the basal metabolic rate; (ii) TH exert a modulatory effect on the brain’s serotonergic system, (iii) general autoimmune/inflammatory mechanisms may contribute to fatigue, and (iv) fatigue may be associated with various comorbidities, including mental disorders, which are more prevalent among hypothyroid patients (9–16).

The association between fatigue and subclinical hypothyroidism remains controversial. Although fatigue is a predominant symptom in subclinical hypothyroidism, its prevalence does not differ significantly from euthyroid individuals and is strongly associated with concomitant diseases (17). Furthermore, several randomized controlled trials indicate that levothyroxine (LT4) treatment for subclinical hypothyroidism in adults does not improve general QoL or alleviate thyroid-related symptoms, including fatigue (18). Therefore, the treatment of subclinical hypothyroidism with TH warrants careful consideration, especially in those individuals with mild elevations of thyroid-stimulating hormone (TSH).

A recent and concerning trend is the lowering of the TSH threshold for initiating thyroid hormone therapy, with as many as 20-30% of patients started on LT4 despite having normal TSH levels (19). One reason behind the tendency to treat euthyroid individuals is the mistaken belief that symptoms commonly linked to hypothyroidism are more dependable for diagnosis than thyroid function tests (20–24). Therefore, exploring the influence of patient-reported fatigue on physicians’ decisions to prescribe TH may be important in understanding the escalating overuse of LT4. The aim of this survey was to explore the impact of patient-reported unexplained fatigue on thyroid specialists’ decisions regarding the prescription of TH.

## 2 Materials and methods

### 2.1 Design

The study is a part of an aggregate data analysis from all national THESIS surveys (Treatment of Hypothyroidism in Europe by Specialists: an International Survey). The THESIS study design and survey details have been previously described in detail (25–29). The project was supervised by a Steering Committee (LH, EVN, EP, PP, RA, RN). Briefly, 26 European countries, each with a population over 4 million, a national endocrine or thyroid professional society, and a national medical journal, participated in the project. Additionally, two Western Asian countries, Turkey and Israel, were included. Participating countries were grouped by geographic region according to the United Nations Statistics Division definition (https://data.un.org/en/index.html): Western Europe (Austria, Belgium, France, Germany, Netherlands, Switzerland), Northern Europe (Denmark, Finland, Ireland, Sweden, United Kingdom), Southern Europe (Croatia, Greece, Italy, Portugal, Serbia, Spain), Eastern Europe (Belarus, Bulgaria, Czech Republic, Hungary, Poland, Romania, Russia, Slovakia, Ukraine), and Western Asia (Israel, Turkey).

The survey targeted practicing specialists, who were members of their respective national endocrine/thyroid societies and managed patients with hypothyroidism. Participants anonymously answered 8 questions regarding their demographics and 23 questions or statements concerning the use of TH in diverse clinical scenarios. The relevant statement regarding fatigue was: “Thyroid hormones may be indicated in biochemically euthyroid patients with unexplained fatigue”. Respondents were asked to select either “yes” or “no.” To further characterize respondents endorsing TH use in euthyroid patients with unexplained fatigue, an additional survey question was re-analyzed regarding potential causes of persistent symptoms in LT4-treated hypothyroid patients achieving normal serum TSH. Respondents expressed their opinion (strongly disagree/disagree/neutral/agree/strongly agree) about potential causes: (i) inability of levothyroxine to restore normal physiology, (ii) psychosocial factors, (iii) comorbidities, (iv) chronic fatigue syndrome, (v) unrealistic patient expectations, (vi) underlying inflammation due to autoimmunity, (vii) burden of chronic disease, and (viii) burden of medication adherence.

### 2.2 Statistics

Data analysis was performed using ‘R’ software. Survey results were not weighted. Categorical (qualitative) variables were presented as frequencies and percentages, while quantitative variables were presented as mean and standard deviation or median and range. Associations between categorical variables were evaluated using Pearson’s chi-square test. Cramer’s V test was used to determine effect size, independent of sample size and p-value. Cramer’s V (ϕc​) values were interpreted according to Rea and Parker: <0.1 (negligible), 0.1-0.2 (weak), 0.2-0.4 (moderate), 0.4-0.6 (relatively strong) and >0.6 (strong association). Multivariate analysis was conducted using logistic regression. Statistical significance was set at p <0.05.

## 3 Results

### 3.1 Baseline characteristics of respondents

Respondent characteristics have been previously described in detail (29). Briefly, survey invitations were distributed to 17,232 members of national thyroid or endocrine societies, with 5695 (33.0%) physicians completing the survey. The majority of respondents were specialists in endocrinology (5132/5695, 90.1%), aged over 40 years (4038/5695, 70.9%), with over 10 years of clinical practice (4487/5693, 78.8%). Most respondents managed more than 100 hypothyroid patients per year (3526/5677, 62.1%), whereas only 2.8% (158/5677) infrequently treated such patients (less than 10 patients per year). Females comprised 65% (3700/5695) of physicians (**Table 1**).

**Table 1:** Characteristics of respondents.

Characteristics	Total N=5695	TH prescribers N= 426 (%)	TH non-prescribers N= 5269 (%)	p value^1^ (Cramer’s V value)^2^
Sex				**p=0.0008** (*ϕ_c_* = 0.04; 95% CI 0.018-0.07)
Female	3700	245 (6.6)	3455 (93.4)	
Male	1995	181 (9.1)	1814 (90.9)	
Age (years)				p=0.17 (*ϕ_c_* = 0.029; 95% CI 0.0-0.05)
≤40	1657	107 (6.5)	1550 (93.5)	
41-50	1565	114 (7.3)	1451 (92.7)	
51-60	1479	124 (8.4)	1355 (91.4)	
>60	994	81 (8.1)	913 (91.9)	
Speciality				**p=0.0014** (*ϕ_c_* = 0.04; 95% CI 0.016-0.07)
Endocrinology	5132	365 (7.1)	4767 (92.9)	
Other	563	61 (10.8)	502 (89.2)	
Hypothyroid patients seen per year^3^				p=0.07 (*ϕ_c_* = 0.035; 95% CI 0.0-0.06)
<10	158	19 (12.0)	139 (88.0)	
10-50	787	51 (6.5)	736 (93.5)	
51-100	1206	82 (6.8)	1124 (93.2)	
>100	3526	274 (7.8)	3252 (92.2)	
Professional experience (years)^3^				p=0.08 (*ϕ_c_* = 0.039; 95% CI 0.026 – 0.065)
≤ 10	1206	77 (6.4)	1129 (93.6)	
11-20	1601	115 (7.2)	1486 (92.8)	
21-30	1476	115 (7.8)	1361 (92.2)	
31-40	1010	76 (7.5)	934 (92.5)	
>40	400	43 (10.8)	357 (89.2)	
Practice at University Centre				p=0.11 (*ϕ_c_* = 0.02; 95% CI 0.0-0.05)
Yes	2181	148 (6.8)	2033 (93.2)	
No	3514	278 (7.9)	3236 (92.1)	
Private Practice				**p=0.003** (*ϕ_c_* = 0.039; 95% CI 0.018 – 0.066)
Yes	1598	146 (9.1)	1452 (90.9)	
No	4097	280 (6.8)	3817 (93.2)	
Member of ETA/ATA/LATS/AOTA^4^				**p=0.003** (*ϕ_c_* = 0.039; 95% CI 0.01-0.06)
Yes	544	58 (10.7)	486 (89.3)	
No	5151	368 (7.1)	4783 (92.9)	

^1^“TH prescribers” *vs* “TH non-prescribers” were analysed using Pearson’s chi-square test. P values <0.05 are in bold.

^2^Cramér’s V is an effect size measurement for the chi-square test of independence [(ϕc values: <0.1 (negligible), 0.1-0.2 (weak), 0.2-0.4 (moderate), 0.4-0.6 (relatively strong) and >0.6 (strong association)].

^3^Some respondents did not answer this question.

^4^ETA, European Thyroid Association; ATA, American Thyroid Association; LATS, Latin American Thyroid Society; AOTA, Asia and Oceania Thyroid Association.

### 3.2 Univariate analysis: characteristics of respondents who considered the use of TH in unexplained fatigue

Of all respondents, 7.5% (426/5695) indicated that TH use may be considered in biochemically euthyroid patients with unexplained fatigue. The likelihood of prescribing TH for unexplained fatigue was significantly higher among respondents who were: (i) male compared to female (9.1% *vs*. 6.6%, p=0.0008, Cramer’s V 0.04, 95% CI: 0.018-0.07), (ii) non-endocrinologists compared to endocrinologists (10.8% *vs*. 7.1%, p=0.001, Cramer’s V 0.04, 95% CI: 0.016-0.07), (iii) members of international thyroid associations compared to non-members (10.7% *vs*. 7.1%, p=0.003, Cramer’s V 0.04, 95% CI: 0.016-0.06) and (iv) specialists working in private practice compared to specialists in public or academic service (9.1% *vs*. 6.8%, p=0.003, Cramer’s V 0.04, 95% CI: 0.019- 0.07). Respondents who infrequently managed hypothyroid patients showed a trend towards affirmative responses, although this difference did not reach statistical significance (**Table 1**).

**Figure 1:**
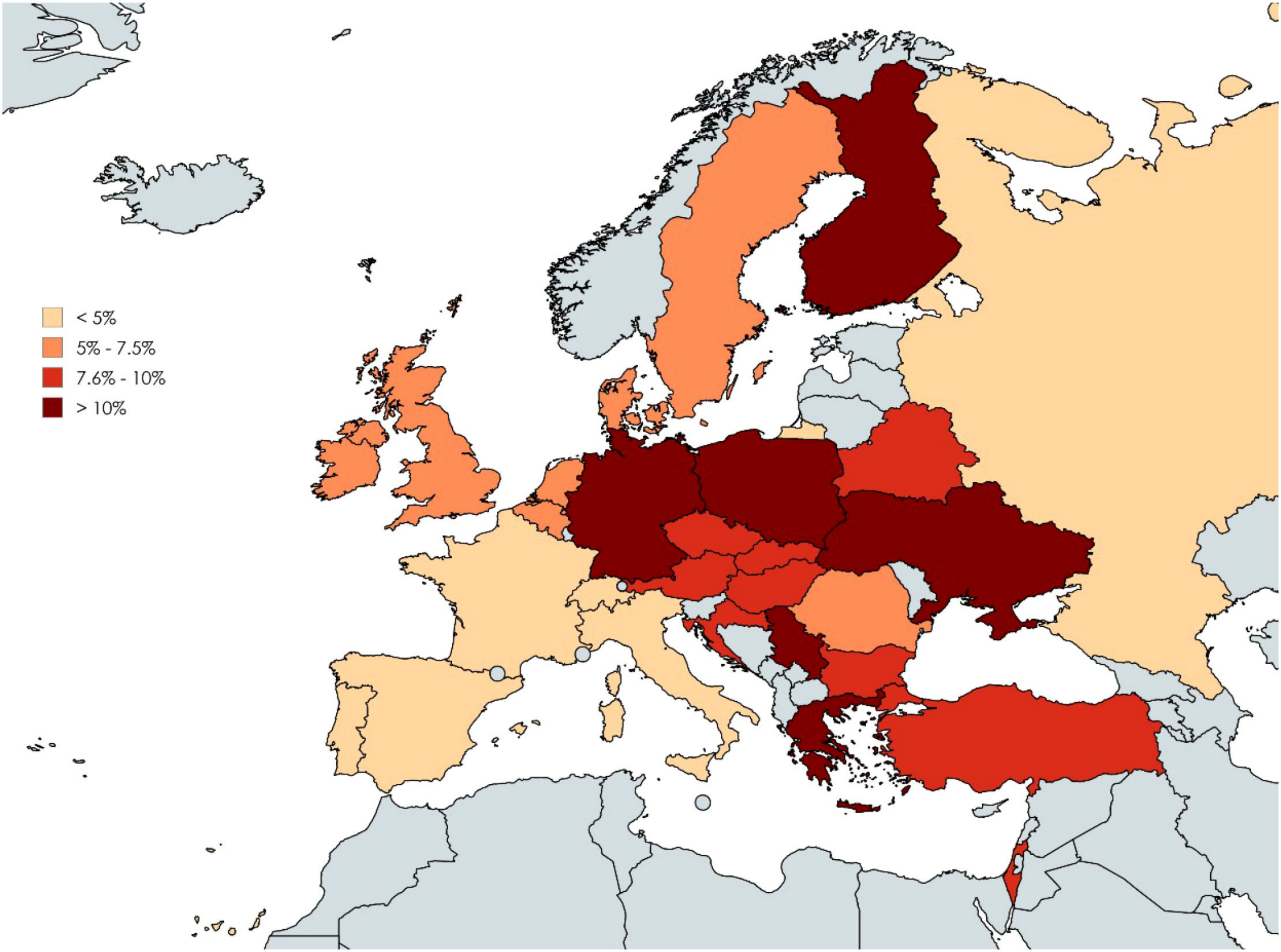
Proportion of respondents considering thyroid hormone (TH) use in biochemically euthyroid patients with unexplained fatigue by country. Color-coding corresponds to percentages of affirmative responses: <5%, 5% - 7.5%, 7.6% - 10% and >10%. Created with MapChart.

The proportion of physicians considering the use of TH in unexplained fatigue varied significantly among geographic regions and countries (p=0.004, Cramer V 0.05, 95% CI: 0.02-0.07, and p=2×10^-16^, Cramer V 0.16, 95% CI: 0.12–0.18, respectively). The lowest percentage of affirmative responses were observed in Western Europe (44/938, 4.7%), while the highest percentages were in Western Asia (27/312, 8.7%) and Eastern Europe (147/1679, 8.8%) (**Table 2**). Fewer than 5% of respondents in Switzerland (1.1%), France (2.8%), Spain (4.1%), Portugal (4.6%), Russia (4.6%), and Italy (4.7%) indicated that TH might be appropriate for unexplained fatigue. Conversely, over 10% of respondents in Ukraine (10.3%), Germany (11.2%), Finland (11.4%), Poland (12.0%), Greece (12.7%), and Serbia (29.3%) considered TH for this indication (**Table 2**, **Figure 1**).

**Table 2:** Number of responders who considered the use of thyroid hormones (TH) in euthyroid patients with unexplained fatigue by region and country.

Region/Country	Responders N	TH prescribers N (%)
Western Europe	**938**	**44 (4.7)**
Switzerland	95	1 (1.1)
France	528	15 (2.8)
Belgium	79	4 (5.1)
Netherlands	35	2 (5.7)
Austria	40	4 (10.0)
Germany	161	18 (11.2)
Northern Europe	**713**	**51 (7.2)**
Denmark	158	8 (5.1)
Ireland	39	2 (5.1)
Sweden	116	7 (6.0)
United Kingdom	277	20 (7.2)
Finland	123	14 (11.4)
Southern Europe	**2053**	**157 (7.6)**
Spain	490	20 (4.1)
Portugal	109	5 (4.6)
Italy	843	40 (4.7)
Croatia	71	7 (9.9)
Greece	441	56 (12.7)
Serbia	99	29 (29.3)
Western Asia	**312**	**27 (8.7)**
Israel	119	9 (7.6)
Turkey	193	18 (9.3)
Eastern Europe	**1679**	**147 (8.8)**
Russia	131	6 (4.6)
Romania	296	15 (5.1)
Belarus	146	11 (7.5)
Slovakia	49	4 (8.2)
Czech Republic	157	14 (8.9)
Bulgaria	120	11 (9.2)
Hungary	160	15 (9.4)
Ukraine	195	20 (10.3)
Poland	425	51 (12.0)

Regions and countries are sorted by the percentage of affirmative responses from low to high. The proportion of affirmative responses to this question varied significantly across countries (p=2 ×10^-16^, Cramer V value 0.16, 95% CI: 0.12 – 0.18).

Bold numbers indicate the numbers of respondents and TH prescribers from the respective regions.

### 3.3 Multivariate analysis: characteristics of respondents who considered the use of TH in unexplained fatigue

Multivariate analysis confirmed positive associations between male gender (p=0.0004, OR: 1.45, 95% CI: 1.18-1.78), practicing in private clinics (p=0.03, OR: 1.27, CI: 1.02-1.58), and practicing outside Western Europe (p=0.006) with more frequent consideration of TH for unexplained fatigue in euthyroid patients. Endocrinologists were less inclined to prescribe TH for this indication than non-endocrinologists (p=0.001, OR: 0.62, 95% CI: 0.46-0.83) (**Table 3**).

**Table 3:** Propensity to prescribe TH in biochemically euthyroid patients with unexplained fatigue (multivariate analysis).

Respondent characteristics	Odds ratio (95% CI)
Endocrinologist	0.62 (0.46-0.83)
Private Practice	1.27 (1.02-1.58)
Male	1.45 (1.18-1.78)
Practice in Northern Europe	1.40 (0.94-2.17)
Practice in Southern Europe	1.60 (1.13-2.27)
Practice in Western Asia	1.83 (1.11-3.02)
Practice in Eastern Europe	1.90 (1.33-2.70)

Geographic regions are compared to Western Europe.

### 3.4 Association between respondents’ view on TH indication for unexplained fatigue and their opinion on potential causes of persistent symptoms

**Figure 2:**
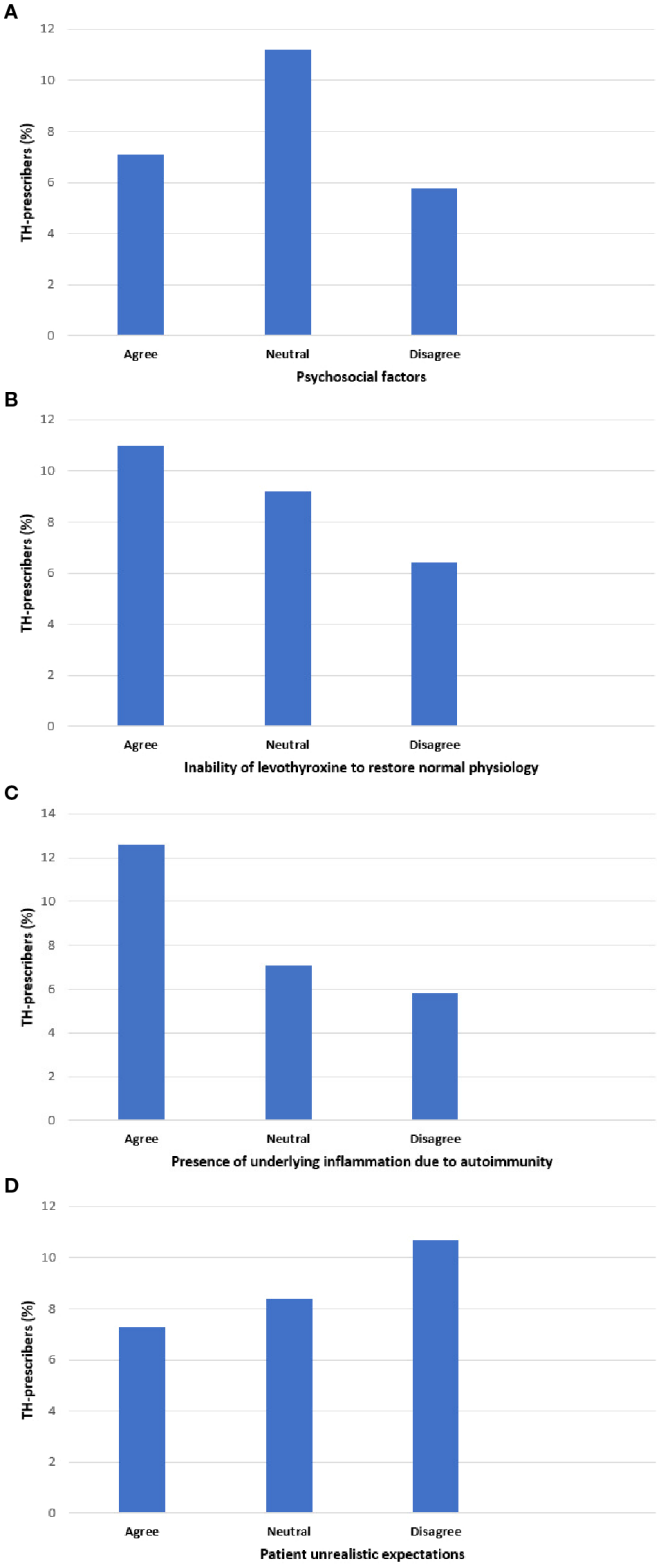
Association between respondent willingness to prescribe thyroid hormones (TH) for euthyroid patients with fatigue, and respondent perceptions about causes of persistent symptoms in hypothyroid patients treated with levothyroxine who have achieved serum TSH levels within the reference range. The horizontal axis shows respondent perceptions for possible causes **(A–D)** of persistent symptoms in hypothyroid patients treated with LT4 who achieve a normal TSH. For each cause **(A–D)**, responses were grouped into three categories: “agree”, “neutral” or disagree. The vertical axis shows the percentage of respondents identified as TH-prescribers for unexplained fatigue. Pearson’s chi-square test: p<0.001.

Participants attributing persistent symptoms to the inability of LT4 to restore normal physiology or underlying autoimmune inflammation were significantly more likely to consider TH for euthyroid patients with unexplained fatigue (p<0.001; Cramer’s V 0.072, 95% CI: 0.045–0.103 and Cramer’s V 0.102, 95% CI: 0.074–0.133, respectively). Respondents attributing persistent symptoms to psychosocial factors were less likely to suggest TH therapy for unexplained fatigue (p<0.001, Cramer’s V 0.067, 95% CI: 0.04–0.098) (**Figure 2**; **Supplementary Table S1**).

## 4 Discussion

To our knowledge, this is the first survey investigating TH use for fatigue of unknown etiology in euthyroid patients. Respondents were predominantly experienced, thyroid-focused physicians from nearly all European countries. A key finding is that a small, yet not negligible percentage of respondents (7.5%) would consider prescribing TH for biochemically euthyroid patients with unexplained fatigue. Considering the high prevalence of fatigue in the general population, such inappropriate use of LT4 therapy may be one of the factors contributing to the escalating overuse of TH in euthyroid individuals (19).

Fatigue is a complex symptom that is reported by healthy individuals and patients with both acute and chronic diseases (30). An estimated 5% to 10% of primary care consultations pertain to fatigue and its highly non-specific nature presents a diagnostic and therapeutic challenge. A systematic review of 26 studies examining the causes of fatigue identified depression as the most frequent diagnosis (18.5%), while underlying somatic diseases (e.g., anemia, diabetes, hypothyroidism, malignancies) accounted for only 4.3% of cases. Myalgic encephalomyelitis/chronic fatigue syndrome was diagnosed in less than 2% of patients (31). Ultimately, an underlying etiology cannot be identified in approximately one-third of cases with fatigue, a situation referred to as medically not yet explained symptoms (MNYES). The management of these patients is a clinical challenge and may lead to inappropriate TH therapy (32–34).

Although the vast majority of respondents follow evidence-based recommendations, it is noteworthy that this survey found that approximately 1 out of 13 European thyroid specialists would consider prescribing TH to biochemically euthyroid patients with unexplained fatigue. Consistent with prior THESIS reports, this management approach exhibits significant variations across geographic regions and countries. Generally, TH use for fatigue was lowest in Western Europe, particularly Switzerland and France (less than 1 out of 30 respondents). Conversely, more than 1 out of 10 respondents in Ukraine, Germany, Finland, Poland, Greece, and Serbia may consider prescribing TH for unexplained fatigue (35–40). In comparison to most European countries, the use of TH in this setting was considered less frequently by specialists in Australia (3%, 2 out of 80), Latin America (2.5%, 2 out of 81), Canada (2.9%, 2 out of 68) and in Japan (2.9%, 6 out of 207) (41–44). The reasons for this discrepancy are at present unknown. It can be speculated that variations in clinical practices across countries or regions may arise from differences in guideline availability, healthcare systems structures, resource allocation and accessibility, patient advocacy, misinformation, training and education standards, as well as cultural influences (23).

Furthermore, the practice of considering TH therapy for fatigue was associated with physician gender (male), specialty (non-endocrinologist), and work setting (private practice). Since the questionnaire did not investigate the rationale for treating these patients, definitive explanations for the observed variations across respondent characteristics cannot be established (26–28, 45, 46). The differences in TH use observed between endocrinologists and non-endocrinologists may reflect greater awareness of the limitations and possible side effects of TH treatment. Further exploration of THESIS data yielded an interesting observation. Respondents’ views on potential causes of persistent symptoms in LT4 treated hypothyroid patients despite normal serum TSH have been previously described in detail (25). Briefly, respondents considered psychosocial factors the primary cause of persistent symptoms. Conversely, underlying inflammation due to autoimmunity and the inability of LT4 to restore normal physiology were considered the least important factors. Respondents attributing persistent symptoms to inability of LT4 to restore normal physiology or underlying autoimmune inflammation were significantly more likely to recommend TH for euthyroid patients with unexplained fatigue. These physicians might wrongly perceive symptoms commonly associated with hypothyroidism as more reliable diagnostic indicators than thyroid function tests. Attributing unexplained fatigue to “clinical hypothyroidism in biochemically euthyroid individuals” may lead to thinking traps and fallacies, including: misdiagnosis, false association, somatic fixation, premature attribution to chronic illness and ultimately in offering an ineffective treatment (47). A randomized double-blind placebo-controlled crossover trial of LT4 in euthyroid patients with symptoms of hypothyroidism demonstrated that TH are no more effective than placebo in improving psychological and physical well-being in such patients (48). Clinicians should instead consider MNYES and adopt a ‘two-track approach” to patient management by giving equal attention to both physical and psychosocial aspects, and, if needed, consulting other health professionals (21, 49, 50).

Several important limitations of this survey warrant discussion. The survey explored TH use for unexplained fatigue via a single statement, lacking any clinical context (e.g., fatigue duration, comorbidities, longitudinal TSH concentrations and anti-thyroid antibody levels). It remains speculative whether TH prescription for fatigue is more common in patients with increasing, “high-normal” TSH levels over time, or very high thyroid peroxidase antibodies levels (51). Although many associations in this study are highly significant, the low Cramer V-values (most often below 0.1) implies that other important drivers for considering TH for euthyroid patients with unexplained fatigue remain to be identified. The strength of this study lie in its large cohort of respondents who regularly manage hypothyroid patients across a wide range of settings (academic centers, public hospitals/clinics, and private practice) and represents nearly all eligible European countries with national endocrine or thyroid societies. Thus, this provides a novel insight into the (mis)use of LT4 and clinical practice in managing fatigue across Europe.

In conclusion, the use of TH in patients with unexplained fatigue may be one of the factors contributing to the escalating overuse of TH. If fatigue relief is the goal of TH treatment in euthyroid subjects, then this approach is likely to result in therapeutic failure, a delay in reaching an appropriate diagnosis and potential harm of overtreatment (including increased risk of cardiovascular morbidity and osteoporosis) (21, 52–54). The results of this study should prompt national endocrine and thyroid associations in countries where TH is frequently recommended for fatigue to validate these findings, explore the underlying drivers, and provide education on the appropriate use of LT4 therapy.

## Data Availability

The datasets presented in this article are not readily available because Data is the property of the THESIS sub-committee. Requests to access the datasets should be directed to TB, tomasz.bednarczuk@wum.edu.pl.

## Supplementary material

Supplementary Table 1 Association between respondents’ view that thyroid hormones may be indicated in biochemically euthyroid subjects with unexplained fatigue (TH prescribers) and potential causes of persistent symptoms in LT4-treated patients with hypothyroidism who achieve normal serum TSH. ^1^ Not all respondents answered the question concerning the cause of persistence of hypothyroid symptoms despite normal serum TSH. ^2^ “TH prescribers” *vs* “TH non-prescribers” were analysed using Pearson’s chi-square test. P values <0.05 are in bold. Cramér’s V is an effect size measurement for the chi-square test of independence. ^3^ The causes of persistent symptoms are ranked from most to least important. ^4^ Answers agree/strongly agree and disagree/strongly disagree were analyzed together.
