# Supplementary material for: Use of thyroid hormones in euthyroid patients with unexplained fatigue: analyses of aggregate data from European national surveys of professional endocrine society members
Source: Front Endocrinol (Lausanne). 2025 Nov 5;16:1713814. doi: 10.3389/fendo.2025.1713814 (PMC12626830; doi:10.3389/fendo.2025.1713814)
Supplement: Supplementary Table 1 — Association between respondents’ view that thyroid hormones may be indicated in biochemically euthyroid subjects with unexplained fatigue (TH prescribers) and potential causes of persistent symptoms in LT4-treated patients with hypothyroidism who achieve normal serum TSH. 1 Not all respondents answered the question concerning the cause of persistence of hypothyroid symptoms despite normal serum TSH. 2 “TH prescribers” vs “TH non-prescribers” were analysed using Pearson’s chi-square test. P values <0.05 are in bold. Cramér’s V is an effect size measurement for the chi-square test of independence. 3 The causes of persistent symptoms are ranked from most to least important. 4 Answers agree/strongly agree and disagree/strongly disagree were analyzed together. [file Table1.docx]

**Supplementary Table 1** Association between respondents’ view that thyroid hormones may be indicated in biochemically euthyroid subjects with unexplained fatigue (TH prescribers) and potential causes of persistent symptoms in LT4-treated patients with hypothyroidism who achieve normal serum TSH.

| **Causes of persistent symptoms^3^** | **Total**  **N**^1^ | **TH prescribers**  **N (%)** | **TH non-prescribers**  **N (%)** | **p value^2^**  **(Cramer’s V value)^2^** |
| --- | --- | --- | --- | --- |
| **Psychosocial factors** | | | | **p< 0.001**  (*ϕ_c_*= 0.067;  95% CI 0.04 – 0.098) |
| Agree^4^ | 3210 | 228 (7.1) | 2982 (92.9) |  |
| Neutral | 597 | 67 (11.2) | 530 (88.8) |  |
| Disagree^4^ | 354 | 42 (11.9) | 312 (88.1) |  |
| **Comorbidities** | | | | p=0.377  (*ϕ_c_*= 0.021;  95% CI 0.021 – 0.053) |
| Agree | 2914 | 226 (7.8) | 2688 (92.2) |  |
| Neutral | 799 | 74 (9.3) | 725 (90.7) |  |
| Disagree | 442 | 37 (8.4) | 405 (91.6) |  |
| **Patient unrealistic expectations** | | | | **p=0.021**  (*ϕ_c_*= 0.043;  95% CI 0.022 – 0.074) |
| Agree | 2559 | 188 (7.3) | 2371 (92.7) |  |
| Neutral | 995 | 84 (8.4) | 911 (91.6) |  |
| Disagree | 606 | 65 (10.7) | 541 (89.3) |  |
| **Chronic fatigue syndrome** | | | | p=0.302  (*ϕ_c_*= 0.023;  95% CI 0.021 – 0.055) |
| Agree | 2478 | 214 (8.6) | 2264 (91.4) |  |
| Neutral | 1136 | 81 (7.1) | 1055 (92.9) |  |
| Disagree | 541 | 43 (7.9) | 498 (92.1) |  |
| **The burden of chronic disease** | | | | p=0.949  (*ϕ_c_*= 0.005;  95% CI 0.021 – 0.028) |
| Agree | 2247 | 185 (8.2) | 2062 (91.8) |  |
| Neutral | 1159 | 92 (7.9) | 1067 (92.1) |  |
| Disagree | 750 | 60 (8.0) | 690 (92.0) |  |
| **The burden of having to take medications** | | | | p=0.836  (*ϕ_c_*= 0.009;  95% CI 0.021 – 0.038) |
| Agree | 1402 | 118 (8.4) | 1284 (91.6) |  |
| Neutral | 1363 | 111 (8.1) | 1252 (91.9) |  |
| Disagree | 1385 | 108 (7.8) | 1277 (92.9) |  |
| **Presence of underlying inflammation due to autoimmunity** | | | | **p< 0.001**  (*ϕ_c_*= 0.102;  95% CI 0.074 – 0.133) |
| Agree | 1148 | 145 (12.6) | 1003 (87.4) |  |
| Neutral | 1430 | 102 (7.1) | 1328 (92.9) |  |
| Disagree | 1574 | 92 (5.8) | 1482 (94.2) |  |
| **Inability of levothyroxine to restore normal physiology** | | | | **p< 0.001**  (*ϕ_c_*= 0.072;  95% CI 0.045 – 0.103) |
| Agree | 981 | 108 (11.0) | 873 (89.0) |  |
| Neutral | 920 | 85 (9.2) | 835 (90.8) |  |
| Disagree | 2260 | 144 (6.4) | 2166 (93.6) |  |

^1^ Not all respondents answered the question concerning the cause of persistence of hypothyroid symptoms despite normal serum TSH.

^2^ “TH prescribers” vs “TH non-prescribers” were analysed using Pearson's chi-square test. P values <0.05 are in bold. Cramér’s V is an effect size measurement for the chi-square test of independence.

^3^ The causes of persistent symptoms are ranked from most to least important.

^4^ Answers agree / strongly agree and disagree / strongly disagree were analyzed together.
